# Reply to ‘Re-evaluating the phylogenetic position of the enigmatic early Cambrian deuterostome *Yanjiahella*’

**DOI:** 10.1038/s41467-020-14922-9

**Published:** 2020-03-09

**Authors:** Timothy P. Topper, Junfeng Guo, Sébastien Clausen, Christian B. Skovsted, Zhifei Zhang

**Affiliations:** 1 Shaanxi Key Laboratory of Early Life and Environments, State Key Laboratory of Continental Dynamics and Department of Geology, Northwest University, 710069 Xi’an, China; 2 Department of Palaeobiology, Swedish Museum of Natural History, Box 50007, SE-104 05 Stockholm, Sweden; 3 School of Earth Science and Resources, Key Laboratory for the study of Focused Magmatism and Giant Ore Deposits, MLR, Chang’an University, 710054 Xi’an, China; 4 Univ. Lille, CNRS, UMR 8198 – Evo-Eco-Paleo, F-59000 Lille, France

**Keywords:** Palaeontology, Taxonomy

## Introduction

**Replying to** Zamora, et al. *Nature Communications* 10.1038/s41467-020-14920-x (2020)

Recently we documented a bilaterally symmetrical, solitary organism, *Yanjiahella biscarpa* from the early Cambrian (Fortunian) of China^1^. We interpreted that *Y. biscarpa* possessed an echinoderm-like plated theca, a muscular stalk similar to hemichordates and a pair of long, feeding appendages. Our interpretation and our phylogenetic analysis suggest that *Y*. *biscarpa* is a stem-echinoderm, which would confirm that echinoderms acquired plates before pentaradial symmetry and that their history is firmly rooted in bilateral forms. Zamora et al.^2^ however, have criticized our interpretation, arguing against an echinoderm affinity, instead suggesting that the phylogenetic placement of *Y*. *biscarpa* is dubious and its significance for understanding deuterostome evolution is uncertain.

This criticism^2^ seems to stem from our interpretation of particular morphological features in *Y*. *biscarpa*^1^ and the perceived lack of echinoderm synapomorphies. Echinoderms possess a calcitic skeleton with a distinctive three-dimensional mesh-like microstructure called stereom, that is considered a major synapomorphy of the Echinodermata^3^. Zamora et al.^2^ highlighted the absence of stereom in *Y*. *biscarpa*, additionally stating that we had omitted appropriate methods, specifically latex casting, that may confirm the presence of stereom in our specimens. We concede that initially we did not latex cast any specimens of *Y*. *biscarpa*, predominantly due to the fragile nature and the associated risk of damaging the specimens in question. In lieu of latex casting we employed Scanning Electron Microscopy (SEM) to investigate the surface and the details of *Y*. *biscarpa* specimens. SEM has been extensively used in the past to study stereom microstructure^4–6^ and if such a microstructure was preserved in *Y*. *biscarpa* it would have been detected using this technique.

**Fig. 1:**
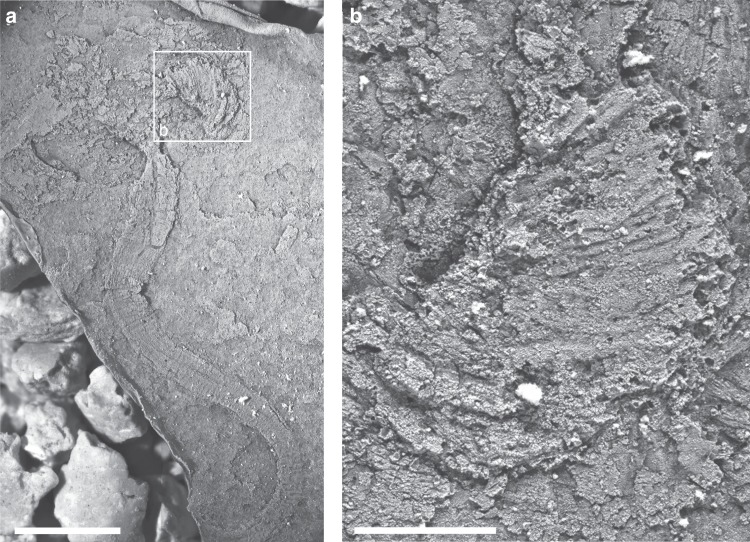
Latex cast of *Yanjiahella biscarpa* from the lower Cambrian Yanjiahe Formation in China. Specimen ELI-HS7A. **a** Specimen with a plated theca and a muscular stalk with mean digestive tract visible, scale bar 4 mm. **b** Detail of plate, with no obvious stereom visible, scale bar 1 mm.

In response to Zamora et al.^2^ we have cast a specimen in latex to investigate whether stereom can be detected in *Y*. *biscarpa*. No pitting, resembling stereom is evident on the plates (Fig. 1) and the latex casts do not appear to offer any further information that was not detectable using light photography and SEM, as predicted. The statement from Zamora et al.^2^ that latex casting could have resolved many of the uncertainties surrounding the composition, shape and arrangement of the plates and the morphology of the stalk is an overstatement and ambitiously delivered without direct examination of the fossil specimens.

The stereom microstructure of Cambrian echinoderms is poorly known^5^, as the majority of specimens are preserved as moulds, or recrystallized skeletons and the primary three-dimensional morphology of their stereom has been obscured^4–6^. Generally, only superficial pitting is preserved and it is this pitting that is interpreted as stereom^7^, typically in specimens that are already recognized or interpreted as echinoderm taxa. Although stereom is a synapomorphy of the Echinodermata, its presence is often presumed and a number of echinoderm taxa have been documented without stereom clearly preserved^8^. We openly state that we cannot observe stereom in the original manuscript and we maintain our stance that this likely is due to taphonomic factors. Zamora et al.^2^ raise additional questions over the interpretation of particular morphological features of *Y*. *biscarpa*. Zamora et al.^2^ highlight that the configuration of the two feeding appendages in *Y*. *biscarpa* differ from previously reported echinoderms, in particular *Ubaghsicystis*, *Dibrachicystis* and *Pleurocystites*. However, these genera are considered to be derived taxa^1,7^ and are not particularly useful in deciphering early echinoderm character traits. As stated in Topper et al.^1^, the precise nature (muscular, collagenous or hydrovascular) of the feeding appendages in *Y*. *biscarpa* is unclear (although their flexibility indicates that they are soft) and consequently without more details we are unable to comment further. Zamora et al.^2^ additionally note that the stalk of *Y*. *biscarpa* is unlike anything seen in the Echinodermata. We appreciated this agreeable statement, as the lack of an analogous stalk in the Echinodermata forms the crux of our argument^1^ morphologically linking *Y. biscarpa* with the hemichordates. Furthermore, Zamora et al.^2^ state that the bilateral symmetry of *Y*. *biscarpa* is poor evidence linking the taxon to the echinoderms. Admittedly we are perplexed by this statement, as we do not use the bilateral symmetrical nature of *Y*. *biscarpa* to support placement within the Echinodermata, but merely to indicate that this interpretation fits with previous studies that report that the early evolution of the group is rooted with bilateral forms^9^.

Zamora et al.^2^ (fig. 2) also re-ran our phylogenetic analysis using a variety of phylogenetic methods and techniques, omitting seven characters that were deemed not parsimony informative. Zamora et al.^2^ also conducted a “thought experiment” in which the presence of stereom was rescored from questionable to absent. Somewhat similar to our results, the analyses do not unequivocally place *Y. biscarpa* within the Echinodermata, but rather as a stem ambulacrarian, a stem hemichordate, or a stem echinoderm, depending on the method employed^2^. These results do not shed any new light on the affinity of *Y*. *biscarpa* and the ambiguity of the trees indicates that there is a large data gap in the early members of each phyla. The “thought experiment”^2^ is also superfluous, as TNT (the program used in our original analyses^1^) scores ambiguous characters as missing^10^. This kind of analysis is frustrated by missing data and highlights the morphological disparity between these two groups. The Bayesian phylogenetic analysis run by Zamora et al.^2^ (fig. 2c) places *Y*. *biscarpa* as a stem echinoderm; however, this position is questioned as the only synapomorphy shared by the group is the possession of plate-like ossicles embedded in the body wall. This synapomorphy is challenged because other Cambrian animals (not included in the analysis), such as the stem entoproct *Cotyledion tylodes*^11^ also have plates/sclerites. The sclerites in *C. tylodes* though are marked by series of tightly spaced lineations that have been considered to represent growth lines and evidence for accretionary growth^11^ and clearly differ from the plates in *Y*. *biscarpa* and all other echinoderm taxa. Zamora et al.^2^ also see similarities in the feeding appendages of *Yanjiahella* with the tentacles of the possible stem deuterostome *Herpetogaster collinsi*^12^, despite the fact that the latter has 2 or 4 highly branching tentacles^12^. The ridges in the proximal stalk are apparently similar to the gill bars in *Oesia disjuncta*^13^; however, the gill bars in *O*. *disjuncta* are U-shaped and show a much greater degree of flexibility^13^. Although Zamora et al.^2^ further dispute an echinoderm affinity based on morphological comparisons with a range of different organisms, these comparisons do not provide a strong counterargument to our interpretation.

As frequently seems to be the case in palaeontology, especially when dealing with early Cambrian organisms, the challenge lies in the interpretation. The early Cambrian is flush with stem group taxa and the dilemma of interpreting the early members of various clades is compounded by the absence of diagnostic features and/or the possession of unfamiliar character combinations. Basal stem group taxa are rare and to some degree barely recognizable because they share few synapomorphies with the crown group in question and it may be expected that only trivial distinctions exist between different stem group taxa at the divergence points leading from a last common ancestor to extant phyla^14^. Zamora et al.^2^ began their reply by listing three characters that define the extant Echinodermata: (1) a calcite skeleton with a mesh-like microstructure (stereom), (2) pentaradial symmetry as adults, and (3) a water vascular system with tube feet. All three characters can be used to define the echinoderm crown group and it appears that the argument presented by Zamora et al.^2^ is based on the assumption that the ancestral member of the Echinodermata should also possess the majority of these crown-group characters. We suggest that more thought needs to be given to how and when these characters were acquired prior to the emergence of the crown group. On the basis of possessing a plated theca, we consider *Y*. *biscarpa* to be a stem echinoderm, inheriting the muscular stalk and linear digestive tract from the last common ambulacrarian ancestor^1^. The degree of phylogenetic resolution in early fossil ambulacrarians is still in its infancy, but the information presented^1^ does give a glimpse of what may be evolving in a concerted manner in the early evolution of the Echinodermata.

In conclusion, we thank Zamora et al.^2^ for their assessment and exploration of the phylogenetic position of *Y*. *biscarpa* and for providing us with the opportunity to further examine and strengthen our original arguments. We believe that their letter and our response will advance the important topic of understanding the early evolution of the Echinodermata. We hope that this undertaking will be the scope of future work in our group and others in the scientific community.

## Methods

### Latex casting

Specimens were cast in latex and coated with ammonium chloride sublimate and photographed using a stereophotographic ZEISS Smart Zoom 5. The fossil material studied herein is deposited at the Early Life Institute of Northwest University, Xi’an (Prefix: ELI).

### Reporting summary

Further information on research design is available in the Nature Research Reporting Summary linked to this article.

## Supplementary information

Reporting Summary

## Data Availability

The authors declare that all data supporting the findings of this study are available within this paper and the original published paper^1^.
