# Supplementary material for: Reply to ‘Re-evaluating the phylogenetic position of the enigmatic early Cambrian deuterostome Yanjiahella’
Source: Nat Commun. 2020 Mar 9;11:1287. doi: 10.1038/s41467-020-14922-9 (PMC7062690; doi:10.1038/s41467-020-14922-9)
Supplement: Supplementary file 1 — Reporting Summary [file 41467_2020_14922_MOESM1_ESM.pdf]

nature research

Corresponding author(s): Timothy Topper

Last updated by author(s): Jan 22, 2020

Reporting Summary

Nature Research wishes to improve the reproducibility of the work that we publish. This form provides structure for consistency and transparency in reporting. For further information on Nature Research policies, see Authors & Referees and the Editorial Policy Checklist .

Statistics

For all statistical analyses, confirm that the following items are present in the figure legend, table legend, main text, or Methods section.

n/a	Confirmed	
☒	☐	The exact sample size (n) for each experimental group/condition, given as a discrete number and unit of measurement
☒	☐	A statement on whether measurements were taken from distinct samples or whether the same sample was measured repeatedly
☒	☐	The statistical test(s) used AND whether they are one- or two-sided<br>*Only common tests should be described solely by name; describe more complex techniques in the Methods section.*
☒	☐	A description of all covariates tested
☒	☐	A description of any assumptions or corrections, such as tests of normality and adjustment for multiple comparisons
☒	☐	A full description of the statistical parameters including central tendency (e.g. means) or other basic estimates (e.g. regression coefficient) AND variation (e.g. standard deviation) or associated estimates of uncertainty (e.g. confidence intervals)
☒	☐	For null hypothesis testing, the test statistic (e.g. F, t, r) with confidence intervals, effect sizes, degrees of freedom and P value noted<br>*Give P values as exact values whenever suitable.*
☒	☐	For Bayesian analysis, information on the choice of priors and Markov chain Monte Carlo settings
☒	☐	For hierarchical and complex designs, identification of the appropriate level for tests and full reporting of outcomes
☒	☐	Estimates of effect sizes (e.g. Cohen's d, Pearson's r), indicating how they were calculated

Our web collection on statistics for biologists contains articles on many of the points above.

Software and code

Policy information about availability of computer code

Data collection	*Provide a description of all commercial, open source and custom code used to collect the data in this study, specifying the version used OR state that no software was used.*
Data analysis	*Provide a description of all commercial, open source and custom code used to analyse the data in this study, specifying the version used OR state that no software was used.*

For manuscripts utilizing custom algorithms or software that are central to the research but not yet described in published literature, software must be made available to editors/reviewers. We strongly encourage code deposition in a community repository (e.g. GitHub). See the Nature Research guidelines for submitting code & software for further information.

Data

Policy information about availability of data

All manuscripts must include a data availability statement. This statement should provide the following information, where applicable:

- Accession codes, unique identifiers, or web links for publicly available datasets
- A list of figures that have associated raw data
- A description of any restrictions on data availability

The authors declare that all data supporting the findings of this study are available within the paper and the original publication

Field-specific reporting

Please select the one below that is the best fit for your research. If you are not sure, read the appropriate sections before making your selection.

☐ Life sciences ☐ Behavioural & social sciences ☒ Ecological, evolutionary & environmental sciences

October 2018

Ecological, evolutionary & environmental sciences study design

All studies must disclose on these points even when the disclosure is negative.

Study description	We analyze the systematic affinity and phylogenetic relationships of Yanjiahella biscarpa from the Yanjiahe Formation in Hubei, China
Research sample	1 specimen
Sampling strategy	Specimens recovered from grey-black, silty shale intercalations in a carbonaceous limestone of the Yanjiahe Formation
Data collection	Specimens were collected by Guo Junfeng
Timing and spatial scale	This part of the Yanijahe Formation is known as Bed III and is Fortunian in age having been correlated with the Purella antiqua shelly fossil zone (Supplementary Figure 1). Carbon isotope chemostratigraphic studies support this age.
Data exclusions	No data has been excluded from the analysis
Reproducibility	*Describe the measures taken to verify the reproducibility of experimental findings. For each experiment, note whether any attempts to repeat the experiment failed OR state that all attempts to repeat the experiment were successful.*
Randomization	This is not relevant to our study
Blinding	This is not relevant to our study

Did the study involve field work? ☒ Yes ☐ No

Field work, collection and transport

Field conditions	Warm and sunny and humid in summer and wet and cold in winter, although the majority of the annual precipitation occurs from May to September.
Location	Gunziao Section, Sandouping, Yichang, Hubei province, China
Access and import/export	Section is accessible by road.
Disturbance	The disturbance at the site has been confined to the rock out crop.

Reporting for specific materials, systems and methods

We require information from authors about some types of materials, experimental systems and methods used in many studies. Here, indicate whether each material, system or method listed is relevant to your study. If you are not sure if a list item applies to your research, read the appropriate section before selecting a response.

Materials & experimental systems

n/a	Involved in the study
☒	☐ Antibodies
☒	☐ Eukaryotic cell lines
☐	☒ Palaeontology
☒	☐ Animals and other organisms
☒	☐ Human research participants
☒	☐ Clinical data

Methods

n/a	Involved in the study
☒	☐ ChIP-seq
☒	☐ Flow cytometry
☒	☐ MRI-based neuroimaging

Palaeontology

Specimen provenance	Gunziao Section, Sandouping, Yichang, Hubei province, China. No permits are required. For more details on locality please see Supplementary Material and the corresponding references
Specimen deposition	The fossil material is deposited at the Early Life Institute of Northwest University, Xi'an (Prefix: ELI).
Dating methods	No absolute dates have been given in the paper. We have given a relative age for the strata which the fossils have been described from. This age has been retrieved using biostratigraphic and isotopic information.

☒ Tick this box to confirm that the raw and calibrated dates are available in the paper or in Supplementary Information.
